# Ultra‐high‐field pharmacological functional MRI of dopamine D1 receptor‐related interventions in anesthetized rats

**DOI:** 10.1002/prp2.1055

**Published:** 2023-02-19

**Authors:** Yuka Kimura, Shunsuke Nakazawa, Kantaro Nishigori, Yuki Mori, Junji Ichihara, Yoshichika Yoshioka

**Affiliations:** ^1^ Drug Development Research Laboratories Sumitomo Dainippon Pharma Co Ltd Osaka Japan; ^2^ Graduate School of Science and Technology, Division of Information Science Nara Institute of Science and Technology (NAIST) Ikoma Japan; ^3^ Center for Information and Neural Networks (CiNet), National Institute of Information and Communications Technology Osaka University Osaka Japan; ^4^ Biofunctional Imaging Laboratory, Immunology Frontier Research Center (IFReC) Osaka University Osaka Japan; ^5^ Present address: Platform Technology Research Unit Sumitomo Pharma Co Ltd Osaka Japan; ^6^ Present address: Global Corporate Strategy Sumitomo Pharma Co Ltd Osaka Japan; ^7^ Present address: Center for Translational Neuromedicine University of Copenhagen Copenhagen N Denmark; ^8^ Present address: Bioscience Research Laboratory Sumitomo Chemical Co Ltd Osaka Japan; ^9^ Present address: Graduate School of Frontier Biosciences Osaka University Osaka Japan

**Keywords:** BOLD, brain imaging, dopamine D1 receptor, pharmacological functional MRI, ultra‐high‐field MRI

## Abstract

The dopamine D1 receptor (D1R) is associated with schizophrenia, Parkinson's disease, and attention deficit hyperactivity disorder. Although the receptor is considered a therapeutic target for these diseases, its neurophysiological function has not been fully elucidated. Pharmacological functional MRI (phfMRI) has been used to evaluate regional brain hemodynamic changes induced by neurovascular coupling resulting from pharmacological interventions, thus phfMRI studies can be used to help understand the neurophysiological function of specific receptors. Herein, the blood oxygenation level‐dependent (BOLD) signal changes associated with D1R action in anesthetized rats was investigated by using a preclinical ultra‐high‐field 11.7‐T MRI scanner. PhfMRI was performed before and after administration of the D1‐like receptor agonist (SKF82958), antagonist (SCH39166), or physiological saline subcutaneously. Compared to saline, the D1‐agonist induced a BOLD signal increase in the striatum, thalamus, prefrontal cortex, and cerebellum. At the same time, the D1‐antagonist reduced the BOLD signal in the striatum, thalamus, and cerebellum by evaluating temporal profiles. PhfMRI detected D1R‐related BOLD signal changes in the brain regions associated with high expression of D1R. We also measured the early expression of c‐fos at the mRNA level to evaluate the effects of SKF82958 and isoflurane anesthesia on neuronal activity. Regardless of the presence of isoflurane anesthesia, c‐fos expression level was increased in the region where positive BOLD responses were observed with administration of SKF82958. These findings demonstrated that phfMRI could be used to identify the effects of direct D1 blockade on physiological brain functions and also for neurophysiological assessment of dopamine receptor functions in living animals.

## Graphical abstract

Dopamine D1 receptor manipulation induced BOLD signal changes in anesthetized rat brain which detected using pharmacological functional MRI. D1‐like receptor agonist increased, while the antagonist decreased the BOLD signal intensity in the striatum, thalamus, and cerebellum. Pharmacological functional MRI can be used for assessment of dopamine receptors‐related neurophysiological actions in live animals.

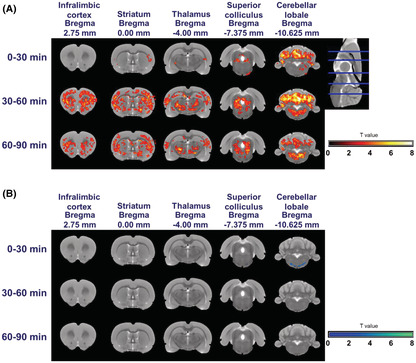

**Abbreviations**
BOLD: blood oxygenation level‐dependent
D1R: dopamine D1 receptor
fMRI: functional magnetic resonance imaging
PFC: prefrontal cortex
phfMRI: pharmacological‐fMRI

## 1 INTRODUCTION

The dopamine system has been getting more and more attention as an important therapeutic target due to its involvement in the pathogenesis of schizophrenia,^1^ Parkinson's disease,^2^ and attention deficit hyperactivity disorder.^3^ To date, dopamine receptor subtypes and two receptor subfamilies (D1‐like and D2‐like receptors) have been defined. Several studies have focused on the role of dopamine D2/D3 receptor (D2R/D3R) subtypes and the dopamine transporter in the pathogenesis of neurological disorders. The current main drug treatments for schizophrenia and Parkinson's disease are D2R/D3R antagonists and agonists, respectively.^4^, ^5^, ^6^ Methylphenidate, which blocks the dopamine transporter leading to an increase in synaptic and extrasynaptic dopamine, is currently the first‐line medication for ADHD.^7^ Although the dopamine D1 receptor (D1R) has been attracting attention as a therapeutic target, the effects of D1R‐specific interventions on each brain region are largely unknown.

D1R is highly expressed in the caudate‐putamen, nucleus accumbens, substantia nigra, and prefrontal cortex (PFC).^8^, ^9^ The receptor was shown to be essential for neurotransmission along the nigrostriatal, mesolimbic, and mesocortical dopamine pathways,^10^ where neurotransmission along these pathways maintains the brain's motor control, working memory, attention, and reward processing functions.^11^, ^12^, ^13^, ^14^ Given this critical role, more insight is needed into the brain‐wide neurophysiological function of D1R.

Functional magnetic resonance imaging (fMRI) is useful to reveal early alterations in brain function. This noninvasive functional imaging technique detects localized changes in oxygenated blood perfusion resulting from the coupling between hemodynamic parameters and neural activity. The image signal changes can be represented as blood oxygenation level‐dependent (BOLD) contrast in T2‐ or T2*‐weighted imaging.^15^, ^16^ Previous clinical and preclinical studies based on experiments mentioned in Lauritzen^17^ have demonstrated that BOLD signal provides a reliable measure of change in neuronal activity. In addition, pharmacological‐fMRI (phfMRI) has found a unique place in drug development. It has been used to assess the neurophysiological functions of particular receptors and evaluate the effects of pharmacological interventions.^18^, ^19^, ^20^ Previously, the value of phfMRI as a noninvasive tool for investigating changes in neurotransmitter function, including dopaminergic neurotransmission, was also demonstrated in living rodent brains.^21^ This study used phfMRI to investigate the direct effects of D1R‐specific interventions on BOLD signal change in anesthetized rodents. The c‐fos expression level was also measured in anesthetized and awake animals to evaluate how anesthesia affects D1R‐related change.

Our study aims were to investigate (1) the neurophysiological modulation induced via D1R and (2) dynamics of D1R‐related change during and after D1R interventions in live anesthetized rats using phfMRI.

## 2 MATERIALS AND METHODS

### 2.1 Animals

All animal procedures were carried out in accordance with the Osaka University Guidelines for Animal Experimentation, the National Institutes of Health Guide for the Care and Use of Laboratory Animals, and the guidelines of the Institutional Animal Care and Use Committee of Sumitomo Dainippon Pharma Co., Ltd. For MR imaging, 21 male Wistar rats (8‐week‐old, Charles River Laboratories Japan, Inc.) were subdivided into three treatment groups: agonist (*n* = 7); antagonist (*n* = 7); and saline (*n* = 7). In addition, to verify our hypothesis that the functional activity level may be lower under isoflurane anesthesia than in an awake state, 21 age‐matched male Sprague–Dawley rats (Charles River Laboratories Japan, Inc.) were subdivided into four treatment groups: saline under awake conditions (*n* = 5); agonist in the awake state (*n* = 5); saline under isoflurane anesthesia (*n* = 5); and agonist under isoflurane anesthesia (*n* = 6). Brain tissue c‐fos expression was measured in each rat. Rats were group‐housed (three/cage) in a temperature‐ (22 ± 2°C) and humidity‐controlled (55 ± 10%) facility under a 12/12 h light/dark cycle and given ad‐libitum access to food and water at least 1 week before experiments.

### 2.2 Compounds

SKF82958 (an agonist of D1‐like receptors; 6‐chloro‐2,3,4,5‐tetrahydro‐1‐phenyl‐3‐[2‐propenyl]‐1H‐3‐benzazepine‐7,8‐diol hydrobromide) was purchased from Sigma‐Aldrich Co. LLC. SCH39166 (an antagonist of D1‐like receptors; [6aS‐trans]‐11‐chloro‐6,6a,7,8,9,13b‐hexahydro‐7‐methyl‐5H‐benzo[d]naphth[2,1‐b] azepin‐12‐ol hydrobromide) was purchased from Tocris Bioscience. The compounds were dissolved to a concentration of 0.6 mg/ml (SKF82958) and 0.2 mg/ml (SCH39166) in physiological saline and were administered subcutaneously at a dose level of 5 ml/kg body weight, based on previous reports.^22^, ^23^ Sterile saline at the same dose was administered for the control procedure.

### 2.3 fMRI experimental protocol

All MRI experiments were conducted using an 11.7 Tesla vertical‐bore Bruker Avance II MR imaging system (Bruker Biospin). All images were acquired using a volume radiofrequency (RF) coil for transmission and reception (m2m Imaging Corp.) and ParaVision 5.1 software (Bruker). Rats were anesthetized with 1%–2% isoflurane (Abbott Laboratories) via a nose cone. The respiratory rate was monitored with an MR‐compatible monitoring system (SA Instruments, Inc.). The fully anesthetized rats were placed in a custom‐made holder and fixed using a tooth bar and adhesive tape to minimize head motion. Subsequently, an infusion cannula was inserted subcutaneously along with an infusion line for compound administration during scanning.

The MRI protocol consisted of: morphological reference imaging using 2D multislice rapid acquisition with a relaxation enhancement sequence (2D‐RARE: repetition time [TR] = 6000 ms, echo time [TE] = 43.9 ms, RARE factor = 16, field of view [FOV] = 3.0 cm × 3.0 cm, slice thickness = 0.5 mm, matrix = 256 × 256 × 48, acquisition time = 12 min); BOLD phfMRI using a multi‐slice RARE (TR = 4166.7 ms, TE = 60 ms, RARE factor = 16, FOV = 3.0 cm × 3.0 cm, slice thickness = 1.0 mm, matrix = 128 × 128 × 24, acquisition time = 5 min/measurement point). PhfMRI images were acquired in a 60‐minute series before subcutaneous infusion of SKF82958 (3 mg/kg, an agonist of D1‐like receptors) or SCH39166 (1 mg/kg, an antagonist of D1‐like receptors), or saline. Afterwards, post‐treatment scanning continued over 90 min. For each animal, the total phfMRI acquisition lasted approximately 150 min.

### 2.4 phfMRI data analysis

#### 2.4.1 Mapping of BOLD signal changes

**FIGURE 1:**
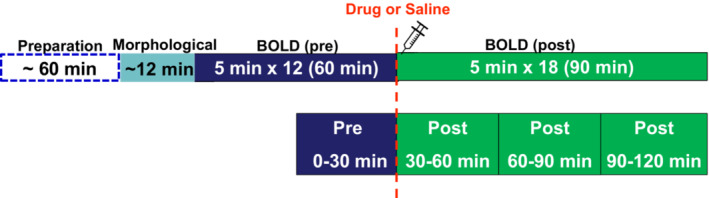
Flow diagram of the MRI protocol used in the study

All MRI data were analyzed using Statistical Parametric Mapping 12 (SPM12) software (The Wellcome Centre for Human Neuroimaging, UCL Queen Square Institute of Neurology, London, UK) in MATLAB 2015a (The MathWorks, Inc.). Skull stripping and brain masking were performed in all morphological images using a rat brain template provided by Tohoku University^24^ (Institute of Development, Aging and Cancer, Tohoku University, Japan; http://www.idac.tohoku.ac.jp/bir/en/db/rb/101028.html [date last accessed 18 November 2015]). After the image preprocessing steps, including realignment, normalization to a template, and smoothing, we applied an “off/on model” to evaluate the statistical differences between the pre‐ and post‐treatment BOLD images. The periods were subdivided into four 30‐min intervals (see Figure 1). Second‐level analysis was performed to generate a t‐contrast map (t‐map) as an fMRI activation contrast pattern. The t‐contrast mapping is a well‐known analysis procedure in fMRI studies to depict neural activation.^25^ A generated t‐map was overlaid on corresponding anatomical brain regions. Group statistical significance for the statistical parametric map was displayed as the t‐contrasts of the BOLD signal changes after each treatment, by comparison of SKF82958 versus saline, and SCH39166 versus saline. Cluster‐level analysis was conducted with statistical significance at *p* < .05 (Family Wise Error [FWE]‐corrected, cluster size >450 pixels, *T* > 2.68), and without correction for multiple comparisons at a number of time points.

#### 2.4.2 Time‐course analysis of the BOLD signal in selected brain regions

The sequential BOLD signal intensities for each animal were measured in the striatum, thalamus, and PFC. The regions were manually segmented in the morphological image by using ITK‐SNAP^26^ (ver. 3.4.0) with reference to the Paxinos–Watson rat brain atlas.^27^ The morphological image and segmented ROIs were overlaid onto respective phfMRI images to depict average BOLD signal changes across time. The BOLD signal was baseline subtracted, and mean BOLD signal was presented at the 0‐, 5‐, 10‐, 15‐, 20‐, and 25‐min time points, respectively.

### 2.5 Brain tissue c‐fos expression measurement

We hypothesized that the functional activity level might be lower under isoflurane anesthesia conditions than in an awake state; therefore, the c‐fos expression level was measured in anesthetized and awake animals subcutaneously injected with SKF82958 (3 mg/kg) or saline. All compounds and kits for RNA analysis, except the RNeasy Mini Kit (Qiagen N.V.), were obtained from Thermo Fisher Scientific Inc. Sixty minutes later after the injection, the rats were sacrificed by decapitation. Striatum, thalamus, and PFC were dissected and frozen at −80°C and fixed in RNA stabilization solution (RNAlater). In each sample, RNA was purified using an RNeasy Mini Kit and quantified with a RiboGreen RNA Quantification Kit. Total RNA (1.0 μg) was converted into cDNA using a High‐capacity cDNA Reverse Transcription Kit. The real‐time quantitative polymerase chain reaction was performed in 20 μl of reaction solution containing cDNA (50 ng), gene‐specific primers for c‐fos (Rn00487426‐g1), 18 S rRNA (T Eukaryotic 18 S rRNA Endogenous Control), and TaqMan Gene Expression Master Mix. The reactions were performed using an Applied Biosystems 7500 Fast Real‐Time PCR system (thermal cycling conditions: 50°C for 2 min, 95°C for 10 min, 95°C for 15 s [40 cycles], and 60°C for 1 min). All collected data were analyzed using the 22DDC T method^28^ and normalized to the housekeeping gene, 18 S rRNA. Relative change of c‐fos expression was calculated by correcting with the average value of the saline control under awake conditions.

### 2.6 Statistical analysis

A two‐way ANOVA followed by post‐hoc evaluation with Bonferroni correction was performed for statistical analyses using GraphPad Prism7 (GraphPad Software Inc.).

### 2.7 Nomenclature of targets and ligands

Key protein targets and ligands in this article are hyperlinked to corresponding entries in http://www.guidetopharmacology.org, the common portal for data from the IUPHAR/BPS Guide to PHARMACOLOGY,^29^ and are permanently archived in the Concise Guide to PHARMACOLOGY 2019/2020.^30^

## 3 RESULTS

### 3.1 D1R activity‐dependent BOLD signal changes

**FIGURE 2:**
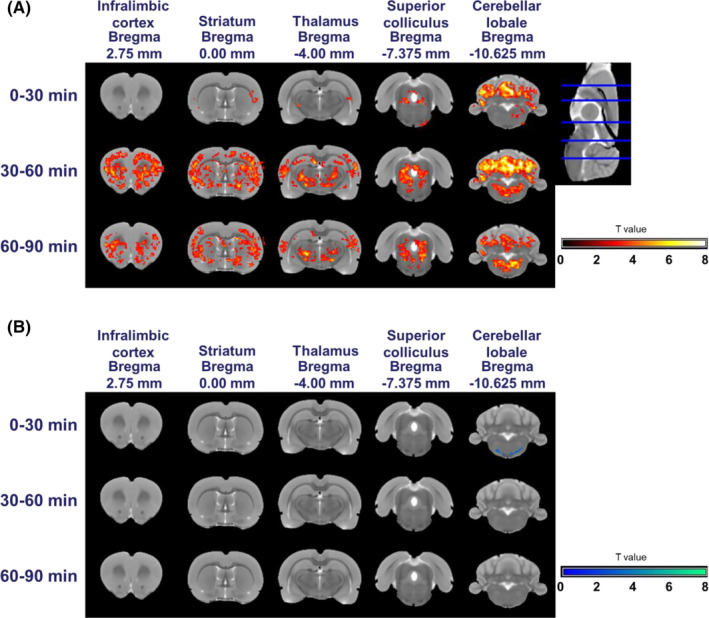
BOLD signal change maps following s.c. injection of the D1‐like receptor agonist, SKF82958. The maps represent the contrast between saline‐treated and SKF82958‐treated animals. The maps in (A) show the regions with increased BOLD signals. Those in (B) show the regions with decreased BOLD signals. Scale bar indicates the random effect *T* values.

**FIGURE 3:**
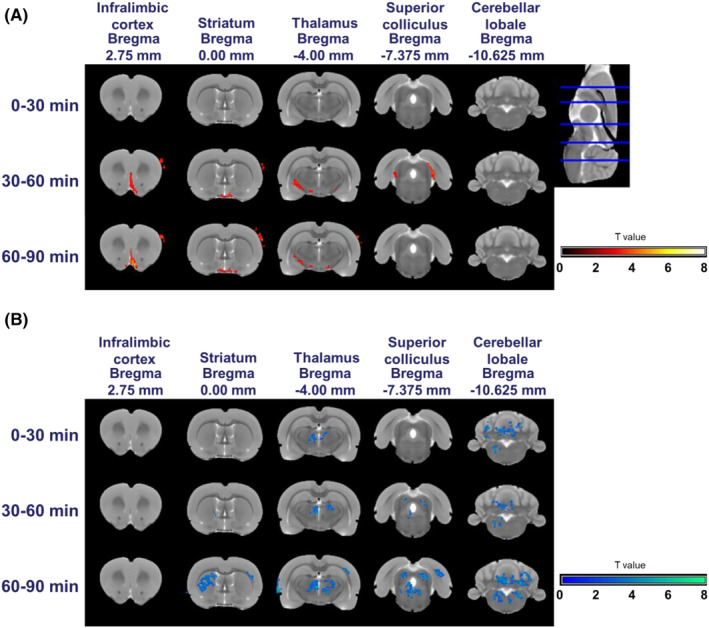
BOLD signal change maps following s.c. injection of D1‐like receptor antagonist, SCH39166. The maps represent the contrast between saline‐treated and SCH39166‐treated animals. The maps in (A) show the regions with increased BOLD signals. Those in (B) show the regions with decreased BOLD signals. Scale bar indicates the random effect *T* values.

BOLD signal change maps represent the effects of SKF82958 or SCH39166 injections (Figures 2 and 3). Overall, both agonist and antagonist compounds induced changes in the BOLD signal compared to saline. Injection of the D1‐like receptor agonist (SKF82958) was associated with an increase in the BOLD signal amplitude in the superior colliculus and cerebellum at 0–30 min after its administration and continuation of this effect until 60–90 min after administration (Figure 2A). SKF82958 also increased the BOLD signal in the infralimbic cortex, striatum, and thalamus at 30–60 min and 60–90 min after administration (Figure 2A). A significant BOLD signal reduction induced by SKF82958 was detectable in the region around the facial nucleus after 30 min (Figure 2B). However, the D1‐like receptor antagonist, SCH39166, reduced the BOLD signal in the thalamus and the cerebellum at 0–30 min after administration. This effect continued until 60–90 min (Figure 3B). We also observed BOLD signal decrement in the striatum and superior colliculus at 60–90 min after administration (Figure 3B). In contrast, a significant increase in BOLD signal was observed in the infralimbic cortex at 30–60 min and 60–90 min after administration (Figure 3A).

### 3.2 Time course of BOLD signal change in selected brain regions

**FIGURE 4:**
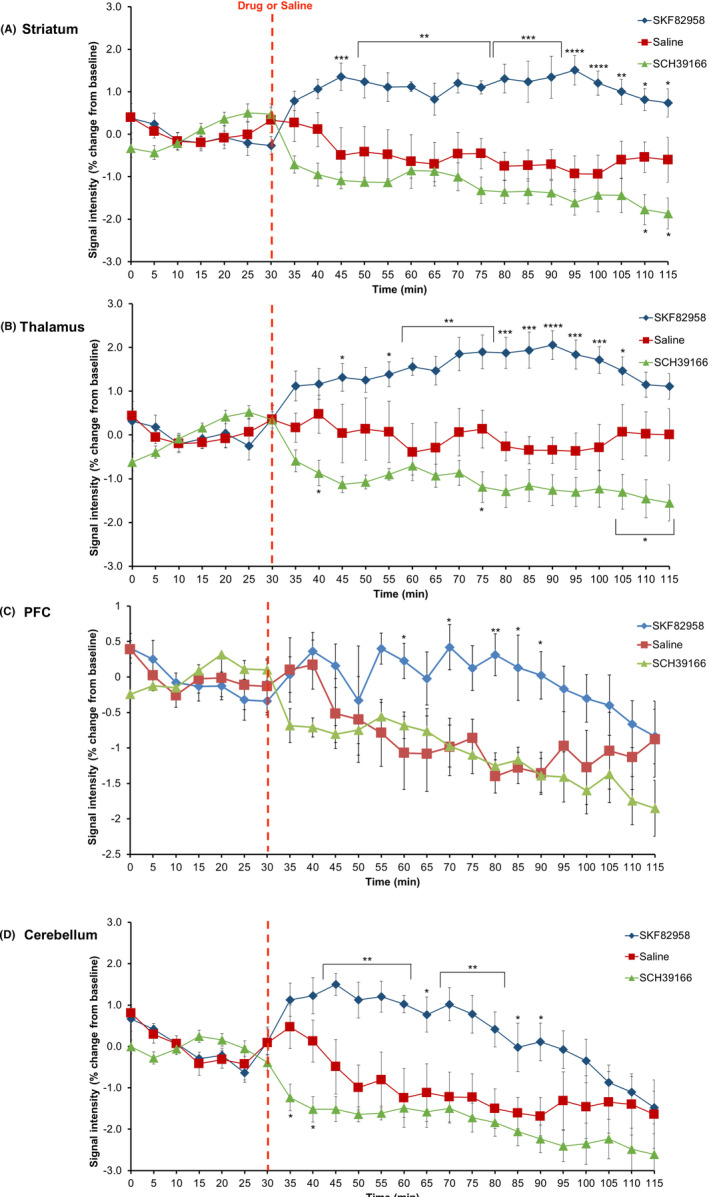
Change in mean BOLD signal intensity during the course of experiment (% from the baseline) in the striatum (A), thalamus (B), PFC (C), and cerebellum (D). Blue line, SKF82958; red line, saline; green line, SCH39166. Data are represented as the mean ± SEM. SEM (*n* = 7). *p < .05, **p < .01, ***p < .001, ****p < .0001 versus saline‐treated group (two‐way ANOVA followed by Bonferroni's multiple comparisons test).

Figure 4 shows the time course of the BOLD signal intensity in the striatum (Figure 4A), thalamus (Figure 4B), PFC (Figure 4C), and cerebellum (Figure 4D). Regardless of ROIs, the overall BOLD signal increased after the administration of SKF82958 and decreased after the administration of SCH39166 (Figure 4). Overall, both agonist and antagonist induced changes in the BOLD signal compared to vehicle.

In the striatum, ANOVA showed significant differences between treatment groups (*F* (2, 18) = 16.61, *p* < .0001), time groups (*F*(17, 306) = 1.99, *p* = .0117), and their interactions (*F*(34, 306) = 3.473, *p* < .0001). In the thalamus, ANOVA showed significant differences between treatment groups (*F* (2, 18) = 17.26, *p* < .0001) and treatment group by time interactions (*F*(34, 306) = 2.624, *p* < .0001), while time group (*F* (17, 306) = 0.7394, *p* = .7614) was not significant. In the PFC, ANOVA showed significant effects of treatment (*F* (2, 18) = 2.885, *p* = .0819), time (*F*(17, 306) = 6.2, *p* < .0001), and their interaction (*F*(34, 306) = 2.05, *p* = .0008). In the cerebellum, ANOVA showed significant effects of treatment (*F* (2, 18) = 7.015, *p* = .0056), time (*F*(17, 306) = 17.57, *p* < .0001), and their interaction (*F*(34, 306) = 3.253, *p* < .0001).

#### 3.2.1 Effects of SKF82958

In the striatum of the SKF82958‐treated brain, signal intensity was significantly higher from 45 min onwards, and this effect remained at 115 min compared to the vehicle group (Figure 4A). In the thalamus, the signal was significantly higher from 55 min and was over by 105 min (Figure 4B). The PFC also showed a significantly higher signal between 60 and 90 min, and the signal gradually returned to baseline (Figure 4C). The trend in the cerebellum was similar, that is, significantly higher between 45 and 90 min with gradual return to baseline (Figure 4D).

#### 3.2.2 Effects of SCH39166

Treatment with SCH39166 briefly showed an opposite trend compared to SKF82958. The BOLD signal in the striatum dropped after treatment and remained significantly lower at 110 and 115 min after treatment compared to the vehicle group (Figure 4A). The signal in the thalamus also showed a similar pattern, with significance seen at 40, 75, 105 min, and onward after treatment (Figure 4B). Although similar trends were observed in the PFC and cerebellum, significance could only be seen at a limited number of time points (Figure 4D).

### 3.3 Effects of SKF82958 on c‐fos expression

**FIGURE 5:**
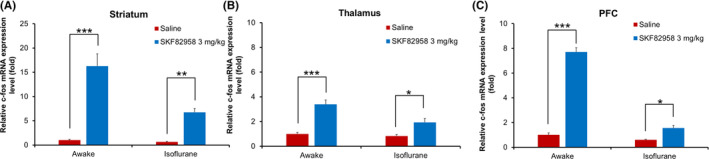
Effects of SKF82958 on c‐fos mRNA expression level under awake conditions or under isoflurane anesthesia conditions, in the striatum (A), thalamus (B), and PFC (C). Data are represented as the mean ± SEM (*n* = 5 or 6). *p < .05, **p < .01, ***p < .0001 versus saline‐treated group (two‐way ANOVA followed by Bonferroni's multiple comparisons test).

We measured the early expression of c‐fos at the mRNA level to evaluate the effects of SKF82958 and isoflurane on neuronal activity. Figure 5 shows the fold change in c‐fos mRNA expression level after SKF82958 treatment over that in the saline control under awake conditions in the striatum (Figure 5A), thalamus (Figure 5B), and PFC (Figure 5C). Regardless of the presence of anesthesia, the D1R agonist increased the c‐fos mRNA expression level in the striatum, thalamus, and PFC. However, the increase in c‐fos mRNA expression provoked by D1R agonist in the striatum, thalamus, and PFC under the awake conditions was attenuated under isoflurane anesthesia conditions. These results indicated that isoflurane decreases the neural activity in brain regions possessing D1 receptors and in brain regions indirectly associated with D1R‐rich areas.

## 4 DISCUSSION

This study investigated the effects of D1R‐specific interventions on BOLD signal change in the brains of anesthetized rats using phfMRI. Our results showed that a D1R agonist and antagonist mainly elicit different BOLD responses in the brain regions such as the striatum, thalamus, infralimbic cortex, and cerebellum, where there are well‐established dopaminergic pathways.^8^, ^9^, ^31^ It has been suggested that these regions maintain motor function, working memory, attention, and reward processing in psychiatric disorders.^11^, ^12^, ^13^, ^14^ Guitart‐Masip et al.^32^ have reported that BOLD variability in subcortical regions is related to the integrity of the dopaminergic system and cognitive function. Our results may reflect the physiological role of D1R. So far, phfMRI has been used to study responses to various specific receptor agonists and antagonists. In terms of the dopamine system, Hagino et al.^33^ have reported that bromocriptine (D2R agonist) had positive BOLD signal changes, while haloperidol (D2R antagonist) had negative BOLD signal changes in specific brain regions such as the hypothalamus where the D2R is present. These results indicated that D2R agonists and antagonists might produce opposite phfMRI responses: an agonist‐induced positive BOLD signal change and an antagonist‐induced negative BOLD signal change. In contrast, few studies have investigated direct responses to D1R agonists and antagonists using phfMRI. As far as we understand, this is the first report of phfMRI used to confirm the BOLD response to D1R regulation. Dixon et al.^34^ have measured changes in regional brain activation following amphetamine administration, either alone or after pretreatment with SCH23390, a D1R antagonist.^34^ Amphetamine has been used widely as a pharmacological challenge agent in human studies and SCH23390 has been shown to attenuate amphetamine‐evoked BOLD activity in the prefrontal cortex, striatum, globus pallidus, thalamus, hippocampus, inferior colliculus, and the cerebellum. Our results correspond to the previously described BOLD responses to D1R modulation in several brain areas, including the striatum, thalamus, infralimbic cortex, and cerebellum. In contrast to the previous report,^34^ our study found no significant effects in the globus pallidus and hippocampus. This discrepancy could be due to differences in experimental protocols. We emphasize that our study specifically focused on direct D1‐like blockade, whereas the previous study focused on D1‐like blockade of amphetamine‐evoked responses, and investigated the nature of the functional response to amphetamine administration.

A previous report compared BOLD fMRI and c‐fos expression measurement using 5‐HT1B/2C receptor agonist m‐chlorophenylpiperazine.^35^ Both methods detected m‐chlorophenylpiperazine‐induced activation in the limbic brain regions that mediate hypophagia, anxiety, and hormone release. Stark et al.^35^ reported a good correlation between brain regions that showed solid c‐fos responses and brain regions that showed positive BOLD responses.^35^ In this study, c‐fos expression level was increased in the region where positive BOLD responses were observed with administration of D1R agonist SKF82958. Conversely, a negative BOLD signal was detected by fMRI in regions where the c‐fos protein was or was not induced.^35^ Negative BOLD signals are controversial, and one common hypothesis of declining BOLD signals is the hemodynamic steal hypothesis, or the blood‐steal hypothesis.^36^, ^37^, ^38^ Our study indicates that the blood steal is unlikely to be a major contributor to negative BOLD signals since none of the negative BOLD regions are adjacent to activated areas. Several studies have shown that areas of negative BOLD responses exhibit reduced oxygenation and metabolism indicative of reduced neuronal activation.^39^, ^40^ They have shown that metabolic downregulation and a local reduction in cerebral blood flow resulted in negative BOLD signals.

In this study, c‐fos mRNA expression studies were performed with Sprague‐Dawley rats because general toxicity studies most often use Sprague‐Dawley rats and the Toxicogenomics project has been constructing a large‐scale database of about 150 compounds using Sprague‐Dawley rats.^41^ The fMRI studies were performed with Wistar rats based on the previous study.^42^ There is no study reporting that D1R agonist/antagonist‐mediated changes in c‐fos expression were similar between the two strains. However, Zamudio et al. have reported about rat strain differences of dopamine receptor levels. They have showed that Wistar rats exhibited a higher level of D1R binding in the basal ganglia subregions than Sprague‐Dawley rats.^43^ These data have suggested that the difference of D1R binding level in these two rat strains may in part relate to D1R‐mediated BOLD activity changes or c‐fos expression changes reported in our study.

The D1‐like receptors, D1R and D5R, share 80% sequence similarity in transmembrane domains^44^; therefore, D1R and D5R are difficult to distinguish pharmacologically by agonists and antagonists. Although the selectivity of SKF82958 for D1R over D5R is not clarified, the previous study, which showed that SKF82958‐induced rotational behavior was blocked by a selective D1R antagonist, SCH23390, indicates that the pharmacological response to SKF82958 was mainly elicited via the D1R.^45^ In addition, the expression of D5R mRNAs was lower than the expression of D1R mRNAs throughout the brain.^46^, ^47^ Thus, the BOLD responses to SKF82958 could be thought to be mainly D1R‐mediated.

D1 and D5 receptor mRNAs are expressed in microvessels of responsive brain areas, so direct effects of dopamine upon the vasculature cannot be ignored in measuring the hemodynamic coupling associated with dopaminergic drugs.^48^ Although Choi et al. suggested that dopamine may respond to D1 receptors expressed in microvessels, we have confirmed activation of neuronal activity in the c‐fos experiment and believe that the response to D1 receptors expressed in neurons has been properly detected.

In this study, we chose isoflurane for anesthesia because it is commonly used in preclinical imaging studies, and it allows easy control of anesthesia depth.^49^ Anesthetics such as isoflurane may influence cerebral blood flow and metabolism via their effects on nitric oxide levels in the brain and thus affect BOLD responses.^50^ In this study, the c‐fos expression level was reduced by 30%–70% in the anesthetized state compared to that in the awake state. In particular, the c‐fos expression level in the PFC was decreased by 70% in the anesthetized state; however, the effect could be evaluated qualitatively even under anesthetized conditions with phfMRI.

In conclusion, this study shows the D1R‐associated BOLD signal changes in the specific brain regions containing high D1R expression. By successfully applying a designed phfMRI protocol, we have demonstrated the applicability of phfMRI to assessment of the dopaminergic brain system and to quantitative scoring of D1R‐mediated neural activation in various brain regions over time. We believe that phfMRI can be used for the in vivo pharmacological assessment of the dopaminergic system and can be further improved as a tool for analyzing pathophysiological conditions such as Parkinson's disease.

## AUTHOR CONTRIBUTIONS

S.N. and K.N. made substantial contributions to the conception of the work. Y.M. and Y.Y. made significant contributions to the data analysis and interpretation. J.I. made significant contributions to the design of the work and the interpretation of data. Y.K. drafted the original manuscript. All authors substantially contributed to the revision of the manuscript drafts. All authors have approved the submitted version of the manuscript and agreed to be accountable for questions related to the accuracy or integrity of any part of the work.

## CONFLICT OF INTEREST

None to declare.

## ETHICAL APPROVAL

All animal procedures were carried out in accordance with the Osaka University Guidelines for Animal Experimentation, the National Institutes of Health Guide for the Care and Use of Laboratory Animals, and the guidelines of the Institutional Animal Care and Use Committee of Sumitomo Dainippon Pharma Co., Ltd. (Osaka, Japan)

## Data Availability

Data available on request from the authors.
